# Screening for gum‐producing Lactic acid bacteria in Oil palm (*Elaeis guineensis*) and raphia palm (*Raphia regalis)* sap from South‐West Nigeria

**DOI:** 10.1002/fsn3.750

**Published:** 2018-09-12

**Authors:** Oniovosa Leonard Adamu‐Governor, Taofik A. Shittu, Oluwatoyin Rebecca Afolabi, Sylvia Veronica Ajagugha Uzochukwu

**Affiliations:** ^1^ Department of Biological Science Yaba College of Technology Yaba Nigeria; ^2^ Department of Food Science and Technology Federal University of Agriculture Abeokuta Nigeria; ^3^ Department of Microbiology Federal University of Agriculture Abeokuta Nigeria; ^4^ Department of Plant Science and Biotechnology Federal University Oye‐Ekiti Nigeria

**Keywords:** gum, Lactic acid bacteria, Oil palm sap, raphia palm sap

## Abstract

Lactic acid bacteria have wide applications in food processing. Lactic acid bacteria produced exopolysaccharides (EPS) which could be used as possible replacer for commercial stabilizer and thickeners produced by nonfood grade bacteria. Seventy‐two samples of Oil and Raphia palm sap were collected in eighteen locations across South‐Western Nigeria and screened for exopolysaccharide production in 6% sucrose agar using streaked plate method. Four hundred EPS‐producing bacteria (EPB) isolated were clustered based on morphological characteristics into two broad groups and preliminary screened for EPS‐producing capacity. Twenty representative of EPB were selected from the broad groups for tentative identification by API 50CHL and 10 high yielding EPB were selected for large‐scale EPS production. Each strain was inoculated into 6% sucrose broth with 3% (v/v) preculture grown overnight in a 1.5 ml flask and incubated at 37°C for 72 hr. The EPSs were purified and freeze‐dried prior to quantification of yields. EPS‐producing bacteria were identified as *Leuconostoc lactis*,* Lactobacillus fermentum*,* Lactobacillus delbrueckii* ssp. *lactis*,* L. delbrueckii* ssp. *delbrueckii*,* Lactobacillus acidophilus*,* Lactobacillus plantarum, Lactobacillus crispatus,* and *Leuconostoc mesenteroides* ssp. *mesenteroides/dextranicum*. EPS yield ranged from 132–810.75 mg/L and EPS‐producing potential of lactic acid bacteria (LAB) strains ranged; 36% (132–245 mg/L), 36% (250–460 mg/L), and 28% (461–820 mg/L). *L. plantarum* had the highest EPS yield of 810.75 mg/L whereas *L. crispatus* had the least yield 242.5 mg/L. These results suggest that majority of LAB in palm wine saps are gum‐producing bacteria. *Leuconostoc* and *Lactobacillu*s were the most abundant LAB found in this study while *L. plantarum* could have applications as potential starter cultures for the production of exopolysaccharides (EPS) at industrial level.

## 1 INTRODUCTION

Lactic acid bacteria (LAB) are gram‐positive and heterogeneous group of bacteria that produce lactic acid as their main fermentation product and are generally recognized as safe (GRAS) (Konings, Kok, Kuipers, & Poolman, 2000; Muñoz, Moreno‐Arribas, & De las Rivas, 2011). LAB are food grade organisms and are important to food and dairy industries, because the lactic acid and other organic acids produced by these bacteria act as natural preservatives and flavor enhancers. LAB are recognized as probiotics which are able to stimulate immune responses and prevent infections against enteropathogenic bacteria (Reid, 1999).

Microbial gums are biodegradable polymers with high molecular weight which are biosynthesized by a wide range of bacteria, yeasts, and fungi (Welman, Maddox & Archer, 2003; Vijayabaskar, Babinastarlin, Shankar, Sivakumar, & Anandapandian, 2011; Welman & Maddox, 2003). Exopolysaccharides are produce by some certain selected microorganisms in the course of fermentation and the resulting exopolysaccharides are usually isolated from the fermentation broth by appropriate procedure (Sutherland, 2001). LAB are well‐known for their ability to produce exopolysaccharide (EPS) (Badel, Bernardi, & Michaud, 2011; Chawla, Bajaj, Shrikant, & Singhal, 2009; De Vuyst & Degeest, 1999; Sutherland, 2001). Lactic acid bacteria produce several types of polysaccharides like many other bacteria that could be classified according to their location relative to the cell. The exopolysaccharide produces by lactic acid bacteria are of two groups; homopolysaccharides and heteropolysaccharides classified based on the composition of their monosaccharides and their biosynthetic pathway (Jolly, Vincent, Duboc, & Neeser, 2002). The microbial ecology of palm wine reveal the presence of bacteria species of the genus *Micrococcus*,* Leuconostoc*,* Lactobacillus,* and *Acetobacter*; while the yeasts usually implicated are *Saccharomyces* and *Candida* spp. (Faparusi & Bassir, 1971; Okafor, 1987; Uzochukwu, Ngoddy, & Balough, 1994). Several LAB have been reported to be responsible for the consistency and soluble white coloration of palm wine through their production of gums, largely dextrans and levans, in the fermentation process of the beverage (Uzochukwu, Ngoddy, & Balough, 1991; Uzochukwu, Ngoddy, Balough, Tucknott, & Lewis, 1994).

According to Hussein, Ibrahim, Asker, & Mahmoud, 2010; EPSs produced by LAB could be used as thickeners, stabilizers, emulsifiers, bodying agents, gelling agents, or fat replacers in several food products and as better alternatives to EPSs produced by Xanthomonas compestris, a pathogen of plants and nonfood grade bacterial. The quantities of EPSs produced by different species and strains of LAB varies between 50 and 350 mg/L depending on growth culture conditions (Cerning, 1995; Ruas‐Madiedo, Hugenholtz, & Zoon, 2002). The low yield of EPS produced by LAB has been attributed to the media composition, conditions of growth, and the method of isolation of EPS. These factors play a key role in media sugar composition and polymer yield (Grobben et al., 1998; Looijesteijn, van Casteren, Tuinier, Doeswijk‐Voragen, & Hugenholtz, 2000; Torino, Sesma, & de Valdez, 2000). The low yields EPSs production by majority of LAB species has reduced their commercial viability and limited industrial applications. Studies have showed that some LAB species have potentials to produce high yields EPSs roughly 40 g/L (Kaditzky & Vogel, 2008; Korakli, Pavlovic, Ganzle, & Vogel, 2003; Minervini et al., 2010). Industrial applications of natural polymers for various uses have increased their demand and this has led to an increased attention toward exopolysaccharides and this has re‐enforced the search for LAB strains with potentials for production of high yields EPS. Exopolysaccharides (EPS) produced by LAB might serve as an excellent source of food grade polysaccharides and as natural alternatives to commercial ones of plant or animal origin.

This study was carried out to identify gum‐producing LAB species and strains responsible for white coloration in palm wine. Isolated LAB will be characterized at phenotypic levels and the determination of strain to help in the selection of autochthonous strains which could be potentially used as starter cultures for the production EPS at the industrial level.

## 2 MATERIALS AND METHODS

### 2.1 Collection of palm sap

Freshly tapped Raphia and Oil palm‐wine were randomly collected in sterile plastic containers from three locations and four palm wine samples from different tappers per location in each of the six states of South‐western Nigeria; Ogun, Oyo, Lagos, Osun, Ondo, and Ekiti. The sterile plastic containers were immediately stored in an ice pack (4°C) and samples were analyzed within a day of collection.

### 2.2 Gum‐producing bacteria isolation

The method described by Uzochukwu et al. (1991) was used to isolate gum‐producing bacteria from palm wine. A loopful of palm wine sample were streaked on 6% sucrose agar and incubated for 24 hr at 35°C. After incubation for 24 hr at 35°C, mucoid colonies were identified as gum‐producing bacteria. Distinct colonies were subcultured severally and pure cultures were stored in 6% sucrose agar slants and stored at 4°C.

### 2.3 Tentative identification of gum‐producing LAB

Pure cultures of gum‐producing bacteria were streaked each on De Man, Rogosa and Sharpe (MRS) agar (Heywood, Lancashire, United kingdom) supplemented with 0.005% cycloheximide (Sigma‐Aldrich) and were incubated at 37°C for 48 hr. Successive subculturing on the same media was done to obtain pure cultures. Pure cultures were stored in sucrose broth containing 15% glycerol (v/v) at −80°C as stock culture. Presumptive LAB were phenotypically characterized by Gram‐staining, cell morphology, and catalase activity by following standard procedures. Only Gram‐positive, catalase negative rod and cocci isolated were selected. Carbohydrate fermentation pattern of lactic acid bacteria were determined in duplicate using API 50CH^®^system (API system, BioMerieux, Mercy ĺ Etoile, France) following the manufacturer's instructions. The results were recorded after 24 and 48 hr of incubation at 37°C and the result interpretation for identification was determined by API Labplus software provided by Bio‐Merieux in Central laboratory, LUTH (Lagos University teaching Hospital, Nigeria).

### 2.4 Microbial gum production

Each of gum‐producing LAB were grown in 50 ml sucrose broth to screen the species/strains of LAB for EPS quantification. Ten representative LAB species/strains with high amount of EPS productivity were selected and used for the production of Exopolysaccharides. 'Cultivation were performed in basal medium (v\v, 6% sucrose, 0.5% peptone, 0.05% K_2_HPO_4_, 0.025% MgSO_4_) in 1.5 L flasks with 1 L working volume. The medium was inoculated with 3% (v/v) overnight culture of isolated bacteria and incubated at 32°C for 72 hr and under noncontrol pH 5.6. Growth was monitored by absorbance measurement at 650 nm using a spectrophotometer (Spectrum lab S23A; Globe Medical, England).

### 2.5 Isolation and quantification of EPS

**Figure 1:**
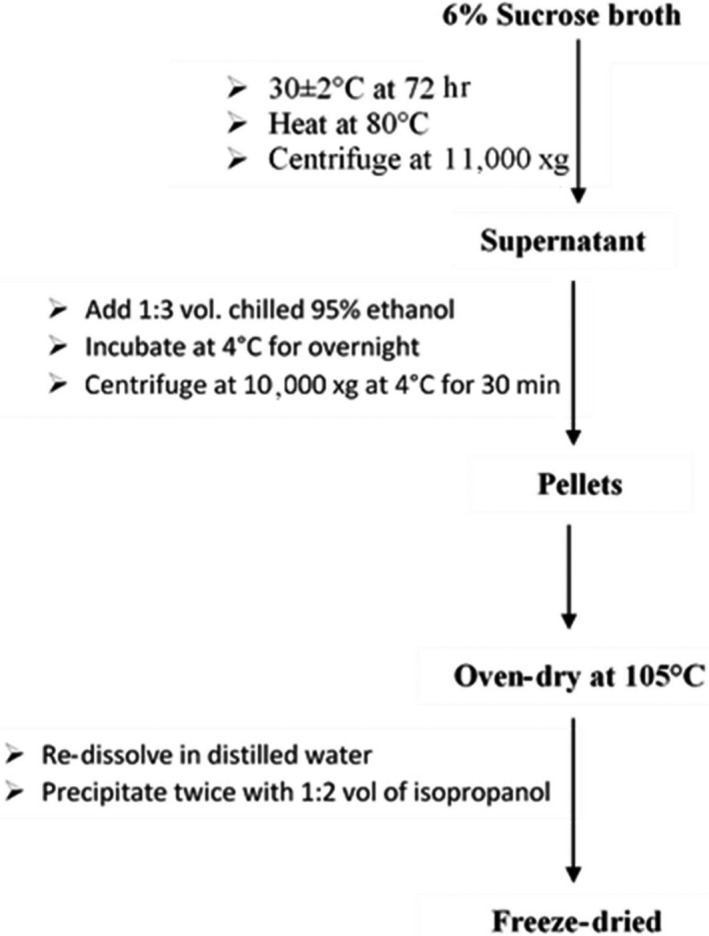
Flow chart of 6% (w/v) sucrose broth for isolation and purification of EPS produced. Source: Ricciardi et al. (2002) and Minervini et al. (2010)

The modified methods described by Ricciardi et al. (2002) and Minervini et al. (2010) were used to isolate and quantify EPS. Modifications included dialysis of EPS with water and isopropanol (Figure 1). Cells were harvested from the fermented culture broth by centrifugation at 11,000×*g* for 30 min in a preweighed tube. Prior to centrifugation, the suspension was stirred with glass rod and heated at 80°C (Heating block) to extract EPS associated to bacteria cells. EPS were precipitated with three volumes of chilled ethanol (95%, v/v) and kept overnight at 4°C for complete precipitation. The precipitated crude was collected by centrifugation at 10,000×*g*, 4°C (J2‐HS; Beckman, USA) for 30 min, and EPS pellets were dried in an oven at 105°C to a constant weight. EPS pellets were dissolved in 50 ml distilled water, precipitated twice with isopropanol and then freeze‐dried (Telster, Cryodos‐8mode, Spain), and quantified by weight. The quantification of EPS was done using dry weight method.

## 3 RESULTS AND DISCUSSIONS

Four hundred (400) gum‐producing bacteria (GPB) were isolated from palm wine samples based on their ability to form mucoid colonies with a glistening and slimy appearance on 6% sucrose agar plates. The formation of mucoid or watery colonies on selected agar media plates and macroscopic detection as the basis of identification of GPB has been reported by several authors (Anbuselvi, Kumar, & Padmaja, 2012; Morin, 1998; Uzochukwu et al., 1991). Four hundred GPB isolates identified from the different palm wine samples were Gram positive, catalase and oxidase negative reactions, thus, considered as presumptive LAB. All GPB isolated from palm wine samples fit the classification of lactic acid bacteria as Gram positive, catalase negative and oxidase negative (Manel, Sana, Nedia, Moktar, & Ali, 2011; Salminen & Von Wright, 1993). The bacteria isolates were cluster into two major broad groups on the basis of their macro‐morphology and micro‐morphology. Ten representative GPB isolates were randomly chosen per group for tentative identification with API 50CHL. Several studies have reported that LAB isolated from palm wine and other fermented food had been initially differentiated on the basis of their cultural and cellular morphology prior to been subjected to various physiological and biochemical tests (Adebayo‐Tayo & Abiodun, 2008; Savadogo et al., 2004).

The identity of twenty gum‐producing bacteria using API 50CHL biochemical profiles is shown in Tables 1 and 2. The result shows that twenty representative GPB isolates were identified as twelve bacteria species belonging to *Lactobacillus* and *Leuconostoc* species. Consequently, 75% belongs to *Lactobacillus* species and 25% *leuconostoc* species. *L. mesenteroides* ssp. *Mesenteroides/dextranicum* (15%) and *L. plantarum* (15%) were the dominant species. The percentage of identification ranged from 76.3% to 99.9%. *Lactobacillus acidophilus* had the lowest percentage identity (76.3%) and *Lactobacillus brevis* had the highest percentage identity (99.9%). The API 50CHL system, which utilizes the characteristics of bacterial sugar fermentation and enzymatic activities, has been commonly used for the identification of lactic acid bacteria (Dickson, Riggio, & Macpherson, 2005; Le Jeune & Lonvaud‐Funel, 1994; Manel et al., 2011). Studies have also reported the dominance of *Lactobacillus* and *Leuconostoc* species in palm wine (Amoa‐Awua, Sampson, & Tano‐Debrah, 2007; Uzochukwu et al., 1991). Earlier studies had reported the presence of *Lactobacillus* and *Leuconostoc* genera in raphia palm wine, coconut palm sap, date palm wine (Atputharajah, Widanapathirana, & Samarajeewa, 1986; Okafor, 1987; Shamala & Streekantiah, 1988). Similarly, Manel et al. (2011) reported *L. mesenteroides* and *L. delbrueckii* as the dominated lactic acid bacteria in Tunisian date palm sap. The capacity of most *Lactobacillus* and *Leuconostoc* strains to produce and secrete large quantities of extracellular enzyme, invertase would enable the utilization and conversion of sucrose into glucose and fructose, and finally organic acids and alcohol (Naknean, Meenune, & Roudaut, 2010) thus enhancing their growth and proliferation over other strains. Uzochukwu et al. (1994), Uzochukwu, Ngoddy, and Balough (1994), Uzochukwu et al. (1999, 2002) and Lasekan, Buettner, and Christlbauer (2007) reported that *Lactobacillus* and *Leuconostoc* strains are responsible for the consistency and soluble white coloration of palm wine through their production of gums, largely dextrans and levans, in the fermentation of the beverage. *Lactobacillus* and *Leuconostoc* species uses sucrose as a sole source of carbon under aerobic and/or anaerobic environment (Valepyn, Berezina, & Paquot, 2012). This could also be an advantage to its proliferation in the sucrose‐rich palm wine sap.

**Table 1:** Identification of Gum‐producing bacteria isolates using API 50CHL

	Isolate code									
	IM 06	IBAii 03	IFEi 05	MOD01	FAT 10	IJE 05	BAD 02	AIY 13	OMO13	MOW 13
LAR	−	−	−	−	−	−	+	+	−	+
RIB	−	+	−	−	−	−	+	−	−	−
DXY	−	+	−	−	−	−	−	−	−	−
GAL	−	+	−	+	−	−	+	−	−	−
MNE	−	+	−	−	−	−	+	−	−	+
RHA	−	−	−	−	−	−	+	−	−	−
MAN	−	−	+	−	−	+	+	−	−	−
SOR	−	−	−	−	−	−	+	−	−	−
MDG	+	−	−	−	−	−	+	−	−	−
NAG	+	+	+	+	+	+	+	+	+	+
AMY	−	−	−	−	−	−	+	−	−	−
ARB	−	−	−	−	−	−	+	−	−	−
ESC	−	−	−	−	+	−	+	−	−	+
SAL	−	−	−	−	+	−	+	−	+	−
CEL	−	−	−	−	−	−	+	−	−	−
MAL	+	+	−	+	+	−	+	+	+	−
LAC	−	−	−	−	−	−	−	+	−	−
MEL	−	+	−	−	−	−	−	+	−	−
TRE	−	+	+	+	+	+	+	−	+	+
MLZ	−	−	−	−	−	−	+	−	−	−
RAF	−	+	−	−	+	−	−	+	+	−
TUR	+	−	−	−	+	−	+	−	+	+
GNT	−	+	−	−	−	−	−	−	−	+
Organism	*Leuconostoc lactis*	*Lactobacillus fermentum*	*Lactobacillus delbrueckii* ssp. *lactis*	*Lactobacillus delbrueckii* ssp. *delbrueckii*	*Lactobacillus acidophilus*	*Lactobacillus delbrueckii* ssp. *lactis*	*Lactobacillus plantarum*	*Leuconostoc lactis*	*L. acidiophilus*	*Leuconstoc mesenter*. ssp. *mesent./dextrac*.

*Note*

+: positive; w: weakly positive; −: negative after 48 hr of incubation at+: positive; w: weakly positive; −: negative after 48 hr of incubation at 37°C. All strains fermented: Glucose, Fructose, N‐Acetylglucosamin and D‐Saccharose, LARA‐L: arabinose; GAL: D galactose; MNE: D mannose; RHA: L rhamnose; MAN: D mannitol; LAC: D lactose; SOR: D sorbitol; MDG: methy‐αD‐glucopyranoside; NAG: N‐Acetylglucosami; TRE: D trehalose; INU: inuline; MLZ: D melezitose; RAF: D raffinose; TUR: D turanose; GNT: potassium gluconate.

**Table 2:** Identification of Gum‐producing bacteria isolates using API 50CHL

	Isolate code									
	IMO 02	ILU 03	IKIii 04	IFE 01	FAT 01	MOD 03	IBA 06	MOW 01	MOW 08	KEL 07
LAR	+	+	+	−	−	+	+	+	−	+
RIB	+	−	+	−	−	−	+	−	+	+
DXY	+	+	+	−	−	−	−	−	−	−
GAL	+	+	+	+	+	−	+	−	+	+
GLU	+	+	+	−	+	+	+	+	+	+
FRU	+	+	+	−	+	+	+	+	+	+
MNE	+	+	+	−	+	+	+	+	+	+
RHA	−	−	−	−	−	−	+	−	+	+
MAN	+	−	+	+	−	−	+	−	+	+
SOR	+	−	−	−	−	−	+	−	+	+
MDG	−	−	−	−	−	−	−	−	+	+
AMY	+	+	+	+	−	−	+	−	+	+
ARB	+	+	+	+	+	−	+	−	+	+
ESC	+	+	+	−	−	+	+	+	+	+
SAL	+	+	+	−	+	−	+	−	+	+
CEL	+	+	+	−	−	−	+	−	+	+
MAL	+	+	+	−	+	−	+	−	+	+
LAC	+	−	+	+	−	−	+	−	−	−
SAC	+	+	+	−	+	+	+	+	+	+
TRE	+	−	+	−	−	+	+	+	+	+
AMD	−	−	−	+	+	−	−	−	−	−
GEN	+	+	+	−	+	−	+	−	−	−
TUR	+	−	+	+	−	+	+	+	+	+
GNT	+	−	+	−	−	+	+	+	−	−
Organism	*Lactobacillus pentosus*	*Lactobacillus coprophilus*	*Lactobacillus brevis*	*Lactobacillus cripatus*	*Lactobacillus delbrueckii* ssp. *delbrueckii*	*Leuconstoc mesenteroides* ssp. *mesent./dextracum*.	*Lactobacillus plantarum*	*Leuconstoc. Mesenteroides ssp mesent./ dextracum*	*Lactobacillus rhamnosus*	*L. plantarum*

*Note*

+: positive; w: weakly positive; −: negative after 48 hr of incubation at+: positive; w: weakly positive; −: negative after 48 hr of incubation at 37°C. All strains fermented: Glucose, Fructose, N‐Acetylglucosamin and D‐Saccharose. LARA‐L, arabinose; GAL: D galactose; GLU: D glucose; FRU: D fructose; MNE: D mannose; RHA: L rhamnose; MAN: D mannitol; LAC: D lactose; SOR: D sorbitol; MDG: methy‐αD‐glucopyranoside; NAG: N‐Acetylglucosamin; AMY: amygdaline; ARB: arbutine; ESC: esculine citrate; SAL: salicine.

The synthesis of exopolysaccharide by gum producing lactic acid bacteria isolated from palm wine is presented in Table 3. The EPS yield production ranged from 242.5–810.75 mg/L. Cultures shown to be the same isolated species did not show similar exopolysaccharide production. The *L. plantarum* strains yield ranged from 677.5 to 810.75 mg/L; *L. delbrueckii* ssp. *lactis* 346.21 to 458.125 mg/L. The highest exopolysaccharide producing isolate strain was *L. plantarum* (810.75 mg/L) whereas, the least exopolysaccharide producing isolate was *L. crispatus* (242.5 mg/L). The values obtained for EPS production and cell dry weight were comparable with data obtained in previous experiments with lactic acid bacteria species/strains (Cerning, 1990; Cerning, Bouillanne, Landon, & Desmazeaud, 1992; Korakli et al., 2003; Minervini et al., 2010; Mozzi, Savoy de Giori, Oliver, & Font de Valdez, 1996; Vogel, 2008). Other studies have also reported that some microorganisms are capable of producing and excreting over 40 g/L of EPSs under conditions of stress (Lin & Cheng‐Chien, 2007; Papinutti, 2010; Ravella et al., 2010). The comparison of EPS yields to cell dry weight results showed that EPS production was growth associated. The growth associated biosynthesis of exopolysaccharides from the selected lactic acid bacteria species/strains was supported by sufficient amount of carbon/nitrogen ratio, and a direct relationship between growth condition (temperature and pH) and exopolysaccharides yields. Sucrose was first converted to glucose and glucose was completely converted into lactic acid to yield the necessary energy, enough nitrogen is necessary for the biosynthesis of essential cell components, while the cells actively produce exopolysaccharides in the presence of an appropriate carbon source (Cerning *et al*., 1994). Growth associated exopolymer production has been observed for several lactic acid bacteria strains (De Vuyst, Vanderveken, Van de Ven, & Degeest, 1998; Ricciardi et al., 2002; Van Den Berg et al., 1995) and other bacteria (Sunil, Amarsinh, Trishala, & Tejswini, 2013; Valepyn et al., 2012). The increase in yields of exopolysaccharides biosynthesis obtained in this study could be attributed to the fermentation temperature and pH. The growth temperature of 32°C and a pH 5.6 was used for the biosynthesis of exopolysaccharides by the selected lactic acid bacteria species/strains. The results obtained in this study are in agreement with previous studies that reported increased exopolysaccharide production at 32 or 37°C compared to a higher growth temperature of 42°C was observed for some *Streptococcus thermophiles* and *L. delbrueckii subspecies bulgaricus* strains (Gancel & Novel, 1994; Mozzi, Oliver, Savoy de Giori, & Font de Valdez, 1995). Increased in EPS yield at low temperatures has also been observed in many LAB (De Vuyst & Degeest, 1999; Minervini et al., 2010; Ricciardi & Clementi, 2000). The optimal pH for EPS production has been found to vary in different strains of LAB (De Vuyst & Degeest, 1999; Ricciardi & Clementi, 2000). Previous studies reported that some *S. thermophiles*,* L. delbrueckii* ssp. *bulgaricus,* and *L. lactis* strains, exopolysaccharide biosynthesis improved considerably when the pH was kept constant at about 6.0 (Cerning, 1995; Mozzi et al., 1996). Other studies have also reported that the optimal pH for EPS production is often close to 6.0 (De Vuyst et al., 1998; Gassem, Schmidt, & Frank, 1997). Similarly, Minervini et al. (2010) reported noncontrolled pH of 5.6 as the optimal pH for the biosynthesis of exopolysaccharides by *Lactobacillus curvatus* DPPMA10. On the contrary, Kumar, Mody, and Jha (2007) reported that there is no single set of culture conditions that guarantees high EPS yields since microorganisms differs in their temperature and pH optima, which are among the critical factors for maximum EPS production.

**Table 3:** Production of EPS in 6% sucrose broth from bacteria isolated from palm wine

S/n	Representative isolate	Microorganism identity	6% Sucrose broth (mg/L)	Cell dry weight (mg/L)
1	IM_a_ 06	*Leuconostoc lactis*	670.61	700.23
2	IBA_b_ 03	*Lactobacillus fermentum*	566.875	540.71
3	IFE_a_ 05	*Lactobacillus delbr*. ssp. *lactis*	458.125	470.43
4	MOD_a_ 01	*Lactobacillus delbrueckii* ssp. *lactis*	346.21	330.95
5	FAT_d_ 01	*Lactobacillus delbr*. ssp. *delbrueckii*	258.125	220.64
6	FAT_a_ 10	*Lactobacillus acidophilus*	351.25	390.98
7	BAD_a_ 02	*Lactobacillus plantarum*	677.5	560.86
8	IFE_c_ 10	*Lactobacillus crispatus*	242.5	240.15
9	MOW_a_ 05	*Leuc mesenteroides* ssp. *mesenteroides*	683.75	610.45
10	BAD_a_ 02	*L. plantarum*	810.75	740.13

*Note*

IM_a_ 06: IMOTA; IBA_b_ 03: IBAFO; IFE_a_ 05: IFE; MOD_a_ 01: MODAKIKI; FAT_a_ 10: FATUNLA; IJE_a_ 05: IJESA ISU‐EKITI; BAD_a_ 03: BADAGARY; AYI_a_ 13: AIYEPE; OMO_a_ 02: OMOTOSHO; MOW_a_ 05: MOWE; ILU_a_ 03: ILUOMOBA; IKI_b_ 04: IKIRE; KEL_b_ 07: KELEB. Subscript a: palm wine tapper one; b: palm wine tapper two; c: palm wine tapper three; d: palm wine tapper four.

Heating treatment of the samples as the first step in the polysaccharide isolation procedure is crucial for completing recovery of the EPS. This could also be the reason for high yield of exopolysaccharides obtained in this study. Studies have shown that heating treatment of the samples as the first step in the polysaccharide isolation procedures resulted in recovery of the exopolysaccharide (Ricciardi et al., 2002; Wang et al., 2010). However, heating treatment should be used only where the exopolysaccharide is thermally stable (Kumar et al., 2007; Rimada & Abraham, 2003). Exopolysaccharides yield generated in this study has shown that the presence of sucrose as a sole carbon source may have had strongly influenced in the total amount of polysaccharide produced. This is in agreement with Stredansky, Conti, Navarini, and Bertocchi (1999) hypothesis that one important factor influencing the yield of synthesized EPS could be the similarity of the substrate to the natural habitat of the microorganisms. Studies have also shown that growth conditions and media composition can affect the quality and quantity of polymer (Mozz et al., 1999; Looijestein et al., 2000; Torino et al., 2000; Smith and de Valdez, 2000, Bramhachari et al., 2007). Studies have also shown that growth conditions and media composition can affect the quality and quantity of polymer (Looijestein et al., 2000; Torino et al., 2000; Smith and de Valdez, 2000, Bramhachari et al., 2007). Other authors have reported that most LAB strains preferred sucrose as their carbon source and in most of these studies an increased microbial growth with corresponding increase in EPS had also been observed (Minervini et al., 2010; Ruas‐Madiedo & de los Reyes‐Gavilan, 2005; Smitinont et al., 1999). However, studies also showed the results are sugar dependence because in each case the most suitable carbohydrate is largely dependent on the strain tested.

## 4 CONCLUSIONS

*Lactobacillus* and *Leuconostoc* species are the dominant gum producing bacteria species in palm wine sap. All 10 strains of LAB selected in this study showed higher yield of EPS that could serve as alternative organisms to produce commercial quantity and *L. plantarum* could be of importance at the industrial level as starter cultures for the production EPS.

## CONFLICTS OF INTEREST

None declared.
